# Managing treatment resistance in schizophrenia: A joint study in Hong Kong and Singapore

**DOI:** 10.3389/fpsyt.2022.1005373

**Published:** 2022-10-20

**Authors:** Shushan Zheng, Sherry Kit Wa Chan, Jimmy Lee

**Affiliations:** ^1^Department of Psychosis, Institute of Mental Health, Singapore, Singapore; ^2^Department of Psychiatry, Li Ka Shing Faculty of Medicine, The University of Hong Kong, Hong Kong SAR, China; ^3^The State Key Laboratory of Brain and Cognitive Sciences, The University of Hong Kong, Hong Kong SAR, China; ^4^Lee Kong Chian School of Medicine, Nanyang Technological University, Singapore, Singapore

**Keywords:** clozapine, treatment resistance, schizophrenia, ECT, clozapine resistance

## Abstract

**Objective:**

This study surveyed clinicians in psychiatry in Hong Kong and Singapore to understand their familiarity and prescribing practices in treatment-resistant schizophrenia (TRS) and clozapine-resistant schizophrenia (CRS).

**Materials and methods:**

All clinicians in psychiatry in both regions were invited through email to participate in an anonymous online survey. The survey collected information on the participants’ characteristics, their familiarity and experience with clozapine use, and their treatment practices in TRS and CRS. Data collection took place between September 2019 and February 2020 in Hong Kong and December 2018 and March 2019 in Singapore.

**Results:**

261 clinicians responded to the survey, with response rates of 19% (105 out of 556 participants) in Hong Kong and 50% (156 out of 309 participants) in Singapore. The majority of respondents (99.0% in Hong Kong; 87.9% in Singapore) were familiar with treatment guidelines for TRS. However, approximately half (54.2% in Hong Kong; 41.7% in Singapore) delayed the prescription of clozapine when indicated. In terms of alternatives to clozapine, approximately half or more of the clinicians in both regions would use high dose antipsychotics, long-acting injectable antipsychotics, antipsychotic polypharmacy, while the adjuvant use of mood stabilizers and electroconvulsive therapy differed between the two regions. In those with CRS, between 10 and 20% of the respondents added adjuvant mood stabilizers or antipsychotics, and 3-10% would use an antidepressant.

**Conclusion:**

Clozapine delays occur in spite of clinicians’ familiarity with treatment guidelines. More research is needed to guide the use of augmentation strategies and the search for effective treatments beyond clozapine.

## Introduction

Schizophrenia afflicts close to 1% of the population (1). Approximately 15–30% of those with schizophrenia develop treatment-resistance (2–4), which is defined as the failure to respond at least two different antipsychotics of adequate dose and duration (5). Clozapine is the only antipsychotic indicated for use in treatment-resistant schizophrenia (TRS; 6). However, 40 to 70% of patients with TRS remain unwell on clozapine and develop clozapine-resistant schizophrenia (CRS; 7, 8).

The quality of life in patients with TRS was thought to be comparable to that of patients with stroke or end-stage renal failure on maintenance dialysis (9). TRS leads to a 10-fold increase in patients’ cost of hospitalizations, health resource utilization, and higher rates of serious co-morbidities (9). Patient with clozapine-resistance have even poorer clinical and functional outcomes than those with TRS (4).

In order to improve recovery outcomes, prompt and effective treatment with clozapine is needed (10). A retrospective chart review of patients with schizophrenia/schizoaffective diagnosis in Toronto, Canada, found that every one-year delay in clozapine initiation increased the odds of long-term clozapine-resistance by 6% (7). Delays in clozapine prescription was also found as a significant predictor of clozapine resistance in a 12-year follow-up study (4). A critical treatment window of 3 years from the onset of TRS was also described, after which the risk of clozapine resistance increases from 30.8 to 81.6% (11).

When clozapine fails, the navigation of treatment choices becomes a challenge due to the scarcity of robust evidence to guide clinicians’ choice beyond clozapine. Studies of clozapine augmentation strategies are often based on open-label trials and meta-analysis of trials that are of low quality (12–17). In today’s clinical practice, the treatment of clozapine-resistance is heterogeneous and determined by the clinician’s experience and the patient’s individual response to the offered intervention.

Understanding clinicians’ practice is the first step in elucidating the gaps that exist in the treatment of patients with TRS. Surveys of clinician attitude toward clozapine prescription have been conducted in various countries, including Denmark (18), India (19), Iran (20), Israel (21), Serbia (22), and the United Kingdom (UK; 23, 24). Most of these studies focused on describing the clinicians’ knowledge and attitudes toward clozapine and their perception of the barriers toward clozapine prescription. Only two of them (18, 19) have described the prescribing practices of the surveyed clinicians in detail. The current study aimed to survey clinicians in Hong Kong and Singapore to understand their practice of managing patients with treatment-resistant schizophrenia. Results from this study pertaining to the clinicians’ experience with clozapine initiation, their perceived barriers to clozapine initiation and factors that might improve clozapine use, were reported in a previous submission (25). This paper focused on understanding the clinicians’ familiarity with antipsychotic treatment guidelines for schizophrenia and explored in detail their treatment approaches in patients with TRS and CRS. Hong Kong and Singapore are both Asian city-states with high urban density that run on a mixed medical economy, where the majority of mental healthcare is delivered by the public sector (26).

## Materials and methods

### Study design

An anonymous online survey was conducted among clinicians in psychiatry in Hong Kong and Singapore to understand their attitudes toward clozapine prescription. The respondents included trainees in psychiatry, resident physicians and psychiatry specialists in both regions. In Hong Kong, the survey invitations were sent through the Hong Kong College of Psychiatrists, the only professional organization for psychiatrists in Hong Kong. All trainees in psychiatry have to register with this organization prior to their specialist training. Therefore, the college has the email addresses of all practicing psychiatric trainees and specialists in Hong Kong. In Singapore, the eligible clinicians and their contact information were obtained from the Singapore Medical Council and National Psychiatry Residency Program, and crossed-checked against the email addresses of psychiatric departments in all restructured hospitals and available emails of private clinics and hospitals. At the time of the study, there were a total of 556 and 309 eligible participants, respectively, in Hong Kong and Singapore. Data collection took place between September 2019 and February 2020 in Hong Kong and December 2018 and March 2019 in Singapore.

Participants were invited *via* email to complete the survey on Questionpro, an online survey platform. The emails explained the purpose of the study and the anonymous and voluntary nature of study participation. Two reminder emails were sent to each participant at separate intervals to encourage their participation in the study.

The survey employed was adapted from Gee et al. (23) in their study of attitudes toward clozapine prescription in practitioners in South London and Maudsley NHS Foundation Trust (23). Participants were asked about their sociodemographic information, the proportion of patients with clozapine under their care, their familiarity with antipsychotic treatment guidelines, their prescribing practices in the treatment of those with TRS, including their threshold for prescribing clozapine as well as the alternatives to clozapine they would use, and their prescription patterns for CRS.

Ethics approval was granted by the relevant ethics review board, i.e., the Institutional Review Board of the University of Hong Kong/Hospital Authority Hong Kong West Cluster in Hong Kong and the National Healthcare Group’s Domain Specific Review Board in Singapore.

### Statistical analyses

The Statistical Package for Social Sciences (SPSS) version 23.0 was used for data analysis. Frequencies and percentages were calculated for categorical variables while mode, median and interquartile range were calculated for ordinal data. Comparisons were performed using corrected chi-square test and Mann-Whitney U test, respectively. Associations were tested using Spearman’s rank correlation. All statistically significant differences were evaluated at the 0.05 level using two-sided tests.

## Results

A total of 261 clinicians from both regions responded to the survey, giving response rates of 19% (105 out of 556 participants) in Hong Kong and 50% (156 out of 309 participants) in Singapore. Missing responses were noted in 1.3 and 0.3% of the total responses provided by the clinicians in Hong Kong and Singapore, respectively. The majority of respondents were male (59.4%), working as psychiatrists (67.4%) in both inpatient and outpatient settings (67.0%), with 6 to 11 years of experience in psychiatry (29.1%). The respondents from both regions had comparable sociodemographic characteristics, as shown in Table 1.

**TABLE 1:** Demographics of participants.

	Total (*N* = 261)		Hong Kong (*N* = 105)		Singapore (*N* = 156)	
	n	%	n	%	n	%
**Gender**						
Male	155	59.4	56	53.5	99	63.5
Female	106	40.6	49	46.7	57	36.5
**Age group**						
23–35 years	114	43.7	47	44.8	67	42.9
36–45 years	83	31.8	30	28.6	53	34.0
46–55 years	34	13.0	12	11.4	22	14.1
>55 years	30	11.5	16	15.2	14	9.0
**Profession**						
Residents or senior residents (i.e., trainees in psychiatry)	81	31.0	28	26.7	53	34.0
Resident physician	4	1.5	NA	NA	4	2.6
Psychiatrists	176	67.4	77	73.3	99	63.5
Current work setting						
In-patient	24	9.2	6	5.7	18	11.5
Out-patient	62	23.8	20	19.0	42	26.9
Mixed	175	67.0	79	75.2	96	61.5
**Years of practice in psychiatry**						
1–5	65	24.9	23	21.9	42	26.9
6–11	76	29.1	28	26.7	48	30.8
11–15	37	14.2	16	15.2	21	13.5
16–20	31	11.9	13	12.4	18	11.5
>20	52	19.9	25	23.9	27	17.3

NA: not applicable.

### Experience and familiarity with treatment guidelines

When asked to provide an estimate of the proportion of patients with clozapine under their care, 72.4% of respondents in Hong Kong and 53.2% of those in Singapore provided a response. Clinicians in both regions reported a median of 5.0% of patients on clozapine under their care (Hong Kong range = 0.3–40.0%, IQR = 3.0–10.0%; Singapore range = 0.0–90.0%, IQR = 1.0–10.0%).

In terms of familiarity with the antipsychotic treatment guidelines in schizophrenia, 1.0% of respondents in Hong Kong and none in Singapore were “not at all familiar” and 12.2% of respondents in Singapore and none in Hong Kong were “a little familiar” with the guidelines. A higher proportion of clinicians in Hong Kong reported being “fairly familiar” (51.4%) and “very familiar” (47.6%) with the guidelines compared to clinicians in Singapore (“fairly familiar” 63.5%, “very familiar” 24.4%; χ^2^_2,261_ = 21.94, *P* < 0.001). The guidelines that the respondents consulted with were similar, including the NICE guidelines, local clinical guidelines and Maudsley Prescribing Guidelines in Psychiatry (in order of increasing familiarity).

Those with more years of practice in psychiatry in Singapore were more familiar with treatment guidelines for TRS (*r* = 0.34, *P* < 0.001). The same association was found in respondents in Hong Kong, but did not achieve statistical significance (*r* = 0.19, *P* = 0.053).

### Prescribing practices in treatment-resistant schizophrenia

When asked about their threshold for clozapine initiation, 45.7% of respondents in Hong Kong and 57.7% of respondents in Singapore reported they would prescribe clozapine after the failure of two antipsychotics. One respondent from Singapore reported starting clozapine early (after one antipsychotic trial), while none of the respondents in Hong Kong would do so. The rest would delay the initiation of clozapine – after the failure of three antipsychotics in 39.0% and 31.4% of respondents in Hong Kong and Singapore, respectively, and after four or more antipsychotics in 15.2% and 10.3% of respondents in Hong Kong and Singapore, respectively.

Those who were more familiar with the treatment guidelines were less likely to delay the initiation of clozapine in both Hong Kong (*r* = –0.21, *P* = 0.03) and Singapore (*r* = –0.17, *P* = 0.03).

As an alternative to clozapine for patients with TRS (see Tables 2, 3), approximately half of the respondents in both regions would ‘often’ or ‘always’ use high dose antipsychotics (55.2% in Hong Kong, 48.1% in Singapore, χ^2^_1,261_ = 1.29, *P* = *0.26*) and long-acting injectable antipsychotics (56.2% in Hong Kong, 50.3% in Singapore, χ^2^_1,260_ = 0.87, *P* = *0.35*). More clinicians in Hong Kong (70.5%) were inclined to ‘often’ or ‘always’ prescribe antipsychotic polypharmacy compared to those in Singapore (47.7%; χ^2^_1,260_ = 13.19, *P* < 0.001). More clinicians in Hong Kong (49.5%) were also inclined to ‘often’ or ‘always’ prescribe adjuvant mood stabilizers compared to those in Singapore (33.5%; χ^2^_1,257_ = 6.60, *P* = *0.01*). In contrast, electroconvulsive therapy (ECT) was more commonly used by Singapore clinicians (56.2%) than those in Hong Kong (24.8%; χ^2^_1,260_ = 25.06, *P* < 0.001).

**TABLE 2:** Answers to questionnaire, shown as percentage of respondents who answered each question (Hong Kong).

On a Likert scale of 1 to 5	1 = “Never”	2	3	4	5 = “Always”	
	n (%)	n (%)	n (%)	n (%)	n (%)	n
**For a patient with treatment resistance, what treatment option would you choose other than clozapine?**						
Antipsychotic polypharmacy	0 (0)	9 (8.6)	22 (21.0)	44 (41.9)	30 (28.6)	105
High dose antipsychotic	4 (3.8)	12 (11.4)	31 (29.5)	40 (38.1)	18 (17.1)	105
Long-acting injectable antipsychotic	3 (2.9)	8 (7.6)	35 (33.3)	43 (41.0)	16 (15.2)	105
Adjuvant mood stabilizer	2 (1.9)	16 (15.2)	35 (33.3)	42 (40.0)	10 (9.5)	105
Electroconvulsive Therapy	10 (9.5)	44 (41.9)	25 (23.8)	23 (21.9)	3 (2.9)	105
**How frequently do you add the following to clozapine as an adjuvant?**						
Antipsychotic	4 (3.8)	36 (34.3)	46 (43.8)	19 (18.1)	0 (0.0)	105
Antidepressant	16 (15.2)	64 (61.0)	22 (21.0)	3 (2.9)	0 (0.0)	105
Mood stabilizer	4 (3.8)	50 (47.6)	30 (28.6)	21 (20.0)	0 (0.0)	105

**TABLE 3:** Answers to questionnaire, shown as percentage of respondents who answered each question (Singapore).

On a Likert scale of 1 to 5	1 = “Never”	2	3	4	5 = “Always”	
	n (%)	n (%)	n (%)	n (%)	n (%)	n
**For a patient with treatment resistance, what treatment option would you choose other than clozapine?**						
Antipsychotic polypharmacy	3 (1.9)	32 (20.6)	46 (29.7)	52 (33.5)	22 (14.2)	155
High dose antipsychotic	7 (4.5)	29 (18.6)	45 (28.8)	61 (39.1)	14 (9.0)	156
Long-acting injectable antipsychotic	5 (3.2)	27 (17.4)	45 (29.0)	63 (40.6)	15 (9.7)	155
Adjuvant mood stabilizer	14 (9.2)	44 (28.9)	43 (28.3)	42 (27.6)	9 (5.9)	152
Electroconvulsive Therapy	7 (4.5)	15 (9.7)	46 (29.7)	70 (45.2)	17 (11.0)	155
**How frequently do you add the following to clozapine as an adjuvant?**						
Antipsychotic	26 (16.7)	62 (39.7)	49 (31.4)	19 (12.2)	0 (0.0)	156
Antidepressant	36 (23.1)	61 (39.1)	44 (28.2)	14 (9.0)	1 (0.6)	156
Mood stabilizer	34 (21.8)	57 (36.5)	44 (28.2)	20 (12.8)	1 (0.6)	156

### Prescribing practices in clozapine-resistant schizophrenia

When patients become clozapine-resistant, 20.0% of respondents in Hong Kong and 13.4% of respondents in Singapore may “often” or “always” add a mood stabilizer; 2.9% in Hong Kong and 9.6% in Singapore may “often” or “always” add an antidepressant; 18.1% in Hong Kong and 12.2% in Singapore may “often” or “always” add an antipsychotic (see Tables 2, 3). While the use of adjuvants was low in both regions, clinicians in Singapore were more inclined to add an antidepressant to augment clozapine compared to clinicians in Hong Kong (χ^2^_1,261_ = 4.46, *P* = 0.04).

## Discussion

The prescribing practices of clinicians in Hong Kong and Singapore in the treatment of patients with TRS and CRS were described in this paper. The findings highlight the existence of clozapine delays in both regions in spite of clinicians’ familiarity with treatment guidelines. Clinicians in the study also have heterogeneous practices when it comes to the offering of non-clozapine alternatives to patients with TRS and their choice of treatment for CRS.

More respondents in Hong Kong (99.0%) and Singapore (87.9%) reported being “fairly” or “very” familiar with treatment guidelines compared to those in original study of practitioner attitudes toward clozapine prescription in South London and Maudsley NHS Foundation Trust (81%; 21). However, there was a longer delay to clozapine prescription by clinicians in our study. 54.2% of clinicians in Hong Kong and 41.7% of clinicians in Singapore would delay the prescription of clozapine. In contrast, close to 80% of practitioners in the London survey initiated clozapine after the failure of two antipsychotic trials as per the guidelines and only 17% of practitioners delayed clozapine initiation (23). Interestingly, the timely manner of clozapine prescription in the South London and Maudsley NHS Foundation Trust appeared to be an exception rather than the norm. The rates of clozapine delay reported in other similar surveys of clinicians’ attitude toward clozapine were closer to the findings of this study. A 2015 study in the UK by Tungaraza and Farooq (24) found that 40.5% of psychiatrists preferred to use several other antipsychotics before considering clozapine. 73% of psychiatrists in an Iranian study likewise preferred other strategies to clozapine when treatment fails with two or three antipsychotics (20). 61.3% of psychiatrists in India would use clozapine in patients who have failed 2 antipsychotics, 17.3% would prefer polypharmacy, 10% would use polypharmacy with clozapine as one of the agents (19). In Denmark, 44.9% of psychiatrists would initiate clozapine after two antipsychotics have failed while 30.6% and 18.4% would wait until three or more than three antipsychotics have failed (18). In Israel, 53.3% of respondents would initiate treatment with clozapine according to the guidelines, while 33% would delay clozapine initiation until the failure of three or more antipsychotics (21).

The delay in clozapine prescription is a common problem across various countries. The barriers to clozapine prescription are often complex and lie beyond a simple lack of knowledge. Our earlier publication (25) suggested that clinicians were deterred from prescribing clozapine due to the need for frequent blood monitoring and concerns about clozapine’s tolerability and its medical complications. Health system factors were also identified as a barrier and clinicians in Hong Kong and Singapore reported a greater need for outpatient resources in terms of clinic and administrative support to improve clozapine prescription (25). These findings were echoed by similar surveys of clinicians in other countries (18–23).

### Alternatives to clozapine

Some patients with TRS may refuse clozapine or fail to tolerate it. In such instances, alternatives to clozapine may be considered. In our study, approximately half of the clinicians in both regions supported the use of long-acting injectable antipsychotics and the use of high dose antipsychotics. The former has been recommended in instances where pseudo-resistance due to non-adherence to antipsychotics is suspected (5). The latter is less grounded in evidence; multiple guidelines have cautioned against the use of supratherapeutic doses of antipsychotics given the higher risk of side effects and little evidence of benefits (27, 28).

Augmentation strategies were commonly adopted by clinicians in both regions. Antipsychotic polypharmacy was prescribed by close to three quarters of clinicians in Hong Kong and half in Singapore, in comparison to the range of 15.9–64.7% reported in other studies (5, 18, 19). Studies that demonstrated the superiority of antipsychotic polypharmacy over antipsychotic monotherapy tended to be open-label trials and low-quality trials, with no superiority showed in double-blinded and high-quality trials (13). Nonetheless, a recent large nationwide cohort study found that those on antipsychotic polypharmacy had an approximately 10% lower relative risk of psychiatric rehospitalization than those on antipsychotic monotherapy, with some antipsychotic combinations working better than others (29).

Approximately half of the clinicians in Hong Kong and one-third in Singapore also employed the use of adjuvant mood stabilizers as an alternative to clozapine monotherapy. A meta-analysis that studied the efficacy of 42 pharmacologic cotreatment strategies added to antipsychotic monotherapy in schizophrenia found significant effect sizes favoring the addition of mood stabilizers, such as lithium and lamotrigine (15). However, the addition of mood stabilizers was ultimately not recommended as the evidence was generally informed by small, short-term trials with poorly reported data (15, 30, 31).

Electroconvulsive therapy (ECT) as an alternative to clozapine was adopted more strongly by clinicians in Singapore than those in Hong Kong. This is in line with the common use of ECT for schizophrenia in Singapore. The Institute of Mental Health is the sole tertiary psychiatric institute in Singapore and sees the majority of patients who are severely psychotic or aggressive in the country (32). It is also the largest center for ECT in Singapore with half of all the performed ECT sessions indicated for schizophrenia (33). Overall, one-third of all ECT sessions in Singapore were indicated for schizophrenia (33). This approach is backed by moderate quality evidence that suggest that ECT augmentation to of a non-clozapine antipsychotic monotherapy is superior to antipsychotic monotherapy alone in the treatment of TRS (34–36).

### Augmentation strategies in clozapine-resistance

In our study, only 10–20% of clinicians would opt to use combination antipsychotics or adjuvant “mood stabilizers” with clozapine. An even smaller number would consider adjuvant antidepressants. In contrast, 76.3% of psychiatrists in a survey in India would combine clozapine with another antipsychotic, with amisulpride being one of the three most preferred agents, followed by risperidone, aripiprazole and haloperidol (19). In a Danish study, when treating resistant-positive symptoms, 39.8% would increase the clozapine dose, 29.6% of respondents would add an atypical antipsychotic, 23.9% would add a conventional antipsychotic, and 4.6% would add a mood stabilizer; when negative symptoms are resistant, 9.5% would increase the clozapine dose, 77.0% would add an atypical antipsychotic, 8.1% an antidepressant and 2.6% a mood stabilizer (18).

In existing literature, the recommendations on augmentation strategies for clozapine non-response have varied across different guidelines (27). This is perhaps a reflection of the lack of robust evidence to support one strategy over the other. The TRRIP Working Group (37) offered the following recommendations on the basis of international expert consensus: Combining clozapine with an antipsychotic, specifically amisulpride or aripiprazole, was suggested for patients who have persistent positive or mixed symptoms or persistent aggression. The combination of clozapine and aripiprazole was found in the earlier-mentioned nationwide cohort study on antipsychotic polypharmacy versus monotherapy to have the best outcome in reducing the risk of rehospitalization, in comparison to clozapine monotherapy as well as other antipsychotic combinations (29). The TRRIP Working Group also recommended the use of adjuvant mood stabilizers (namely, lithium, and lamotrigine) in patients with persistent aggression and/or suicidal ideation, and adjuvant antidepressants in patients with persistent negative symptoms or suicidal ideation (37). While the use of ECT in CRS was not studied in our survey, ECT shows promise and was mentioned by the TRRIP Working Group as a possible treatment for those with persistent positive symptoms, mixed symptoms or suicidal ideation (36–38).

### Strength and limitations

This is the first survey that described the insights on experience, familiarity and prescribing practices in the treatment of patients with TRS and CRS in clinicians from Hong Kong and Singapore. The response rates of 19% and 50% in Singapore in Hong Kong were comparable to the average response rates in previous survey studies from the same regions (39, 40). There was a good completion of the survey questions by the respondents, with missing responses in 1.3% and 0.3% of the total responses in Hong Kong and Singapore, respectively. The similar sociodemographic characteristics of respondents in both regions also allowed for a more meaningful comparison of the survey responses. However, the small sample size for the Hong Kong participants might have led to false negatives in some of the results. The management approach of TRS in this study was limited to the use of pharmacotherapy and ECT, and did not include the use of psychological interventions such as cognitive behavioral therapy for psychosis. Psychological interventions are gaining ground as complementary treatment in psychosis, though the evidence-base for their use remains limited and more research in this field is still needed (12, 41–43). Other limitations of this study include the possibility of recall bias and social desirability bias due to the self-reporting nature of the survey. Selection bias may also be present as the respondents were more likely to be clinicians who were experienced in the use of clozapine and those who were concerned about its underutilization.

## Conclusion

Clozapine is the only evidence-based treatment in patients with TRS. However, its initiation across the world is often delayed and this delay in turn leads to higher incidences of clozapine-resistance and poorer outcomes for patients (4). There is a need to examine and address the factors that are preventing the timely prescription of clozapine in order to facilitate its earlier adoption. More research into the biological mechanisms that underpin treatment-resistance and clozapine-resistance in schizophrenia is needed in order to guide the search for effective treatments beyond clozapine.

## Data availability statement

The original contributions presented in this study are included in the article/supplementary material, further inquiries can be directed to the corresponding author.

## Ethics statement

The studies involving human participants were reviewed and approved by The Institutional Review Board of the University of Hong Kong/Hospital Authority Hong Kong West Cluster in Hong Kong and the National Healthcare Group’s Domain Specific Review Board in Singapore. The patients/participants provided their written informed consent to participate in this study.

## Author contributions

All authors listed have made a substantial, direct, and intellectual contribution to the work, and approved it for publication.
